# Antimicrobial resistance in *Neisseria gonorrhoeae* among men who have sex with men: a 10-year analysis with implications for doxycycline post-exposure prophylaxis

**DOI:** 10.1128/jcm.00965-26

**Published:** 2026-09-25

**Authors:** Phyu Mon Latt, Ei T. Aung, Nyi Nyi Soe, Norelle L. Sherry, Ivette Aguirre, Vesna De Petra, Fabian Y. S. Kong, Eloise Williams, Paula Roydhouse, Eric P. F. Chow

**Affiliations:** 1 Melbourne Sexual Health Centre, Bayside Health 198098 , Melbourne, Victoria, Australia; 2 School of Translational Medicine, Faculty of Medicine, Nursing and Health Sciences, Monash University 2541, Melbourne, Victoria, Australia; 3 Microbiological Diagnostic Unit Public Health Laboratory, Department of Microbiology & Immunology, The University of Melbourne at the Peter Doherty Institute for Infection and Immunity, Melbourne, Victoria, Australia; 4 Department of Infectious Diseases & Immunology, Austin Health 3805, Heidelberg, Victoria, Australia; 5 Centre for Pathogen Genomics, University of Melbourne 2281, Melbourne, Victoria, Australia; 6 Centre for Epidemiology and Biostatistics, Melbourne School of Population and Global Health, The University of Melbourne 2281, Melbourne, Victoria, Australia; 7 Victorian Infectious Diseases Reference Laboratory, Royal Melbourne Hospital at the Peter Doherty Institute for Infection and Immunity, Melbourne, Victoria, Australia; 8 Department of Infectious Diseases, The University of Melbourne at the Peter Doherty Institute for Infection and Immunity, Melbourne, Victoria, Australia; Marquette University, Milwaukee, Wisconsin, USA

**Keywords:** MSM, gonorrhea, antimicrobial resistance, antibiotics, sexually transmitted infections, prevention, sexually transmitted diseases, HIV, doxyPEP

## Abstract

The implementation of HIV pre-exposure prophylaxis and doxycycline post-exposure prophylaxis may influence antimicrobial resistance in *Neisseria gonorrhoeae* among men who have sex with men (MSM). We examined trends in antimicrobial resistance among MSM, stratified by HIV and PrEP status, and assessed tetracycline resistance in relation to doxyPEP use. We included all MSM with an *N. gonorrhoeae* isolate at Melbourne Sexual Health Centre (MSHC) between 2015 and 2024. Resistance was defined using 2025 EUCAST breakpoints and epidemiological cutoff values. Trends were assessed using the Cochran-Armitage test, and tetracycline resistance was compared between doxyPEP users and non-users using the chi-square test. A total of 11,334 isolates from 6,611 MSM were included. Ceftriaxone resistance remained rare (3/11,334; 0.03%). The proportion of isolates with acquired azithromycin resistance mechanisms increased from 0.9% (8/940) in 2015 to 6.4% (102/1,584) in 2024 (*P*_trend_ < 0.001), above the 5% level at which the WHO recommends that an antimicrobial no longer be used for empirical treatment. Ciprofloxacin resistance increased substantially, from 18.5% (170/920) to 55.2% (875/1,584) (*P*_trend_ < 0.001), with significant trends in all three groups. Tetracycline resistance, assessable from 2019 onward, was consistently high (around 80%), with no significant trend. Among 1,020 isolates with known doxyPEP status, tetracycline resistance was significantly higher in doxyPEP users than in non-users (89.2% [107/120] vs 77.2% [695/900], *P* = 0.003). High-level tetracycline resistance (MIC > 8 mg/L) was also more common among doxyPEP users (54.2% vs 29.7%, *P* < 0.001). Ceftriaxone remains effective for empirical gonorrhea treatment among MSM in Melbourne. Rising the proportion of azithromycin non-wild-type raises concerns about its ongoing utility in dual therapy. Higher tetracycline resistance among doxyPEP users, particularly high-level tetracycline resistance, underscores the need to monitor for multidrug-resistant strains as doxyPEP use expands.

**IMPORTANCE:**

Antimicrobial-resistant *Neisseria gonorrhoeae* is an urgent global health threat, and the expanding use of doxyPEP among MSM has raised concerns regarding its selective pressure on resistance. Linking a decade of individual-level clinical data to over 11,000 isolates from a major Australian sexual health clinic, we show that the proportion of azithromycin non-wild-type has risen to 6.4%, surpassing the 5% threshold at which the WHO recommends discontinuing an agent for empirical therapy. This finding raises concerns about the ongoing utility of dual ceftriaxone-azithromycin treatment. Furthermore, tetracycline resistance was significantly higher among doxyPEP users, with a disproportionate increase in high-level resistance (MIC >8 mg/L) that was associated with ciprofloxacin co-resistance. These findings provide timely real-world evidence to inform gonorrhea treatment guidelines and antimicrobial surveillance as doxyPEP implementation expands globally.

## INTRODUCTION

Gonorrhea, caused by *Neisseria gonorrhoeae*, remains a major public health concern globally, with an estimated 82.4 million new infections among adults aged 15 to 49 years worldwide in 2020 (1). In Australia, gonorrhea diagnoses have more than doubled over the past decade, with 44,210 cases reported in 2024 (2). *N. gonorrhoeae* has a remarkable capacity to develop resistance to antimicrobials used for its treatment (3). Both the World Health Organization (WHO) and the United States (US) Centers for Disease Control and Prevention have classified antimicrobial-resistant *N. gonorrhoeae* as an urgent public health threat, and it is listed as a priority organism in the Australian National Antimicrobial Resistance Strategy (4, 5).

Currently, dual therapy with ceftriaxone and azithromycin is the recommended first-line treatment for uncomplicated gonorrhea in Australia (6), while the WHO and several countries, including the USA and the United Kingdom, have moved to ceftriaxone monotherapy (7–9). However, strains with resistance to ceftriaxone and acquired resistance mechanisms to azithromycin have been reported internationally, raising concerns about the future of effective gonorrhea treatment (10, 11).

Men who have sex with men (MSM) are disproportionately affected by gonorrhea and may be at particular risk of antimicrobial-resistant infections due to higher rates of sexually transmitted infections (STIs) and frequent antibiotic exposures (12, 13). Since 2016, the large-scale HIV pre-exposure prophylaxis (PrEP) programs in Australia have been accompanied by frequent STI screening and high detection of asymptomatic STIs among MSM (14, 15). More recently, doxycycline post-exposure prophylaxis (doxyPEP), taken within 72 h after condomless sex, has shown high efficacy in reducing incident syphilis and chlamydia, and more modest, setting-dependent effects on gonorrhea in clinical trials (16). However, there are concerns that increased doxycycline use may increase the prevalence of multidrug-resistant *N. gonorrhoeae*, as plasmid-mediated tetracycline resistance determinants are frequently linked to resistance to other antimicrobials (17, 18). In 2023, the Australasian Society for HIV, Viral Hepatitis and Sexual Health Medicine (ASHM) recommended doxyPEP primarily for syphilis prevention among gay, bisexual, and other MSM, while noting continuing uncertainty about potential harms, including increased antimicrobial resistance (AMR) (19). The impact of HIV PrEP-associated antibiotic exposures and doxyPEP on gonococcal AMR remains unclear.

We previously examined trends and risk factors for antimicrobial-resistant *N. gonorrhoeae* at the Melbourne Sexual Health Centre (MSHC) from 2007 to 2018 (20). However, that study included only 1 year of data following the widespread implementation of HIV PrEP in Australia, did not stratify by HIV PrEP use, and preceded the introduction of doxyPEP. In this study, we aimed to examine the proportion and trends of AMR in *N. gonorrhoeae* among MSM from 2015 to 2024, stratified by HIV status and PrEP use, and to assess resistance patterns in relation to doxyPEP use.

## MATERIALS AND METHODS

### Study setting, data sources, and definitions

The MSHC is the largest publicly funded sexual health clinic in Victoria, Australia. The center provides approximately 50,000 consultations per year, and approximately 40% of clients are men who have sex with men (MSM) (21). Clients’ demographic and behavioral data are collected through a computer-assisted self-interview (CASI) at the initial visit and at any subsequent visits at least 3 months apart, as part of routine clinical care. Prior to December 2023, CASI was completed via touchscreen kiosks at the clinic, and from December 2023 onward, MSHC transitioned to a Digital Front Door (DFD) system, allowing clients to complete CASI on their personal smartphones (22). Data from CASI are linked to the clinic’s electronic medical system, the Clinic Practice Management System (CPMS), from which study data were extracted. A question on doxyPEP use in the past month was added to the CASI from December 2023 onward. As CASI is completed every 3 months, the doxyPEP status was not captured at every consultation.

We extracted data from the CPMS for all MSM who had a positive *N. gonorrhoeae* culture with minimum inhibitory concentration (MIC) testing performed between 1 January 2015 and 31 December 2024. Variables included age, country of birth, Indigenous status, sex worker status, sexual contact with someone from overseas in the preceding 12 months, HIV status, HIV pre-exposure prophylaxis (PrEP) use, and doxycycline post-exposure prophylaxis (doxyPEP) use. Antimicrobial susceptibility data were obtained from the Microbiological Diagnostic Unit Public Health Laboratory (MDU PHL) at the University of Melbourne, which operates the on-site laboratory at MSHC.

Each isolate was assigned to one of three mutually exclusive groups based on the individual’s HIV and PrEP status at the time the isolate was collected: people living with HIV (PLHIV), HIV-negative PrEP users, and HIV-negative non-PrEP users. Individuals whose status changed during the study period therefore contributed isolates to more than one group.

### Antimicrobial susceptibility testing

All MSM attending MSHC were offered nucleic acid amplification test (NAAT) screening for *N. gonorrhoeae*. Individuals with a positive NAAT result were recalled to the clinic for culture. Culture was also performed at the initial visit for individuals presenting with symptoms. All *N. gonorrhoeae* isolates were confirmed based on Gram stain, oxidase, and matrix-assisted laser desorption ionization-time of flight mass spectrometry (MALDI-TOF MS). Minimum inhibitory concentrations (MICs) were determined for ceftriaxone, azithromycin, ciprofloxacin, and tetracycline using the agar dilution reference method.

AMR was defined using the 2025 European Committee on Antimicrobial Susceptibility Testing (EUCAST) clinical breakpoints, and epidemiological cutoff values (ECOFFs) were applied where clinical breakpoints were unavailable (23). Ceftriaxone resistance was defined by EUCAST as MIC >0.125 mg/L. Additionally, isolates with MIC = 0.125 mg/L were flagged as having decreased susceptibility, consistent with the WHO and Australian Gonococcal Surveillance Programme reporting thresholds, given their potential clinical significance (10). For azithromycin, EUCAST has not established clinical breakpoints, but isolates with MICs >1 mg/L were classified as having acquired resistance mechanisms (non-wild-type) based on the ECOFF, which marks the upper limit of the wild-type MIC distribution. The ECOFF identifies isolates that have acquired a resistance mechanism, but it does not predict clinical treatment failure. Non-wild-type proportions are therefore not directly equivalent to clinical resistance. Ciprofloxacin resistance was defined as MIC >0.06 mg/L; isolates with MICs between 0.03 and 0.06 mg/L were classified as “susceptible, increased exposure” and were not included in the resistant category. Tetracycline resistance was defined as MIC >0.5 mg/L. The resistant isolates were further categorized as low level (MIC 1–8 mg/L) or high level (MIC >8 mg/L) (24). Tetracycline MIC data were available from 2015 onward. However, due to breakpoint and methodology changes over this time period, isolates tested between 2015 and 2018 had MICs recorded as ≤4 mg/L, which did not allow classification as susceptible or resistant according to the EUCAST breakpoint (>0.5 mg/L). Therefore, tetracycline resistance was only assessed from 2019 onward.

### Statistical analyses

Demographic characteristics were reported once per individual, using their record at their first visit during the study period, as reporting them at the isolate level would count individuals with multiple isolates more than once. AMR analyses were based on all isolates tested, with HIV and PrEP status assigned at the time each isolate was collected.

Continuous variables were summarized as medians with interquartile ranges (IQR), and categorical variables as frequencies and percentages. The proportion of AMR was calculated for ceftriaxone, ciprofloxacin, and tetracycline. For azithromycin, the proportion of isolates with acquired resistance mechanisms (MIC >1 mg/L, above the ECOFF) was calculated. All proportion estimates are reported with 95% confidence intervals (CIs) estimated using the exact binomial method. Trends in AMR over time were assessed using the Cochran-Armitage test for trend, overall and stratified by HIV/PrEP status, for each antimicrobial.

For visits without a valid doxyPEP response, only “Yes” or “No” responses were carried forward from the nearest previous visit for the same participant within a 90-day window. This window matches the CASI completion interval, so the carried-forward response represents the most recent information available for that participant. Responses were never carried backward from a later visit. The proportion of tetracycline-resistant isolates was compared between doxyPEP users and non-users using the Pearson’s chi-square test.

Data analyses were performed using Stata version 19 (StataCorp, College Station, TX, USA), and figures were generated using R (R Foundation for Statistical Computing, Vienna, Austria).

## RESULTS

### Study population and demographic characteristics

Between 1 January 2015 and 31 December 2024, a total of 11,334 *N. gonorrhoeae* isolates were tested for MICs from 6,611 MSM attending MSHC. Of these, 719/6,611 (10.9%) were PLHIV, 2,063/6,611 (31.2%) were HIV-negative PrEP users, and 3,829/6,611 (57.9%) were HIV-negative non-PrEP users. The median age at first visit was 29 years (IQR 25–35). The majority of individuals were born in Oceania (51.6%), followed by Southeast Asia (12.3%) and the Americas (9.4%). The proportion of PrEP users increased substantially over the study period, from 0.6% (13/2,063) in 2015 to 21.2% (438/2,063) in 2024 (*P*_trend_ < 0.001)*,* reflecting the large-scale rollout of PrEP in Australia from 2016 onward (Table 1).

**TABLE 1:** Demographic and clinical characteristics of men who have sex with men with *Neisseria gonorrhoeae* isolates at Melbourne Sexual Health Centre, 2015–2024^*b*^

Variable	All individuals (*n* = 6,611)	People living with HIV (*n* = 719)	HIV-negative, PrEP users (*n* = 2,063)	HIV-negative, non-PrEP users (*n* = 3,829)
Age, median (IQR)				
Median	29 (25–35)	34 (29–41)	31 (27–36)	28 (24–33)
Year, *n* (%)				
2015	709 (10.7%)	91 (12.7%)	13 (0.6%)	605 (15.8%)
2016	549 (8.3%)	44 (6.1%)	38 (1.8%)	467 (12.2%)
2017	708 (10.7%)	79 (11.0%)	101 (4.9%)	528 (13.8%)
2018	725 (11.0%)	75 (10.4%)	173 (8.4%)	477 (12.5%)
2019	711 (10.8%)	80 (11.1%)	208 (10.1%)	423 (11.0%)
2020	435 (6.6%)	72 (10.0%)	146 (7.1%)	217 (5.7%)
2021	512 (7.7%)	65 (9.0%)	238 (11.5%)	209 (5.5%)
2022	730 (11.0%)	70 (9.7%)	365 (17.7%)	295 (7.7%)
2023	738 (11.2%)	73 (10.2%)	343 (16.6%)	322 (8.4%)
2024	794 (12.0%)	70 (9.7%)	438 (21.2%)	286 (7.5%)
Country of birth, *n* (%)				
Oceania	3,412 (51.6%)	361 (50.2%)	1,083 (52.5%)	1,968 (51.4%)
Southeast Asia	813 (12.3%)	122 (17.0%)	238 (11.5%)	453 (11.8%)
Americas	620 (9.4%)	86 (12.0%)	223 (10.8%)	311 (8.1%)
Northwest Europe	509 (7.7%)	37 (5.1%)	159 (7.7%)	313 (8.2%)
Northeast Asia	423 (6.4%)	51 (7.1%)	139 (6.7%)	233 (6.1%)
Southern and Central Asia	273 (4.1%)	21 (2.9%)	53 (2.6%)	199 (5.2%)
Southern and Eastern Europe	175 (2.6%)	15 (2.1%)	51 (2.5%)	109 (2.8%)
North Africa and the Middle East	113 (1.7%)	12 (1.7%)	33 (1.6%)	68 (1.8%)
Sub-Saharan Africa	90 (1.4%)	7 (1.0%)	30 (1.5%)	53 (1.4%)
Missing	183 (2.8%)	7 (1.0%)	54 (2.6%)	122 (3.2%)
Current sex worker, *n* (%)				
Yes	27 (0.4%)	4 (0.6%)	13 (0.6%)	10 (0.3%)
No	3,031 (45.8%)	357 (49.7%)	926 (44.9%)	1,748 (45.7%)
Decline to answer	121 (1.8%)	42 (5.8%)	31 (1.5%)	48 (1.3%)
Missing	3,432 (51.9%)	316 (43.9%)	1,093 (53.0%)	2,023 (52.8%)
Sex with someone from overseas in the preceding 12 months, *n* (%)				
Yes	794 (12.0%)	76 (10.6%)	279 (13.5%)	439 (11.5%)
No	1,939 (29.3%)	232 (32.3%)	557 (27.0%)	1,150 (30.0%)
Unknown/missing	3,878 (58.7%)	411 (57.2%)	1,227 (59.5%)	2,240 (58.5%)
Indigenous status, *n* (%)				
Aboriginal and/or Torres Strait Islander	92 (1.4%)	11 (1.5%)	23 (1.1%)	58 (1.5%)
Neither	5,869 (88.8%)	656 (91.2%)	1,840 (89.2%)	3,373 (88.1%)
Missing	650 (9.8%)	52 (7.2%)	200 (9.7%)	398 (10.4%)
Sampling site, *n* (%)				
Oropharynx	2,771 (24.4%)	265 (17.8%)	978 (22.3%)	1,528 (28.0%)
Urethra	3,747 (33.1%)	438 (29.4%)	1,373 (31.4%)	1,936 (35.4%)
Anorectum	4,814 (42.5%)	787 (52.8%)	2,025 (46.3%)	2,002 (36.6%)
Others^*a*^	2 (0.0%)	1 (0.1%)	1 (0.0%)	0 (0.0%)

^*a*^ Others included wound swab (anatomical site unclear) and sites not stated.

^*b*^ All demographic characteristics are reported per individual at the first visit. Sampling site data represent all specimens collected (*n *= 11,334), as multiple anatomical sites may be sampled from a single individual.

Isolates were obtained from the anorectum (42.5%; 4,814/11,334), urethra (33.1%; 3,747/11,334), and oropharynx (24.4%; 2,771/11,334). Anorectal isolates were most common across all three groups, representing a particularly high proportion among PLHIV (52.8%, 787/1,491) and PrEP users (46.3%, 2,025/4,377), compared with non-PrEP users (36.6%, 2,002/5,466). Full demographic and clinical characteristics are presented in Table 1.

### Trends in AMR between 2015 and 2024

Ceftriaxone resistance remained rare throughout the study period (3/11,334; 0.03%). The three resistant isolates came from three different individuals: one anorectal isolate detected in 2019 (MIC 0.5 mg/L) and two urethral isolates detected in 2023 (both MIC 0.25 mg/L). All three were also resistant to ciprofloxacin and tetracycline. The two isolates detected in 2023 additionally had high-level azithromycin resistance (MIC >256 mg/L), rendering them resistant to all four antimicrobials tested. A further 11 isolates from nine individuals had decreased susceptibility to ceftriaxone (MIC = 0.125 mg/L); 10 were also resistant to both ciprofloxacin and tetracycline, bringing the total with MIC ≥0.125 mg/L to 14 (0.12%).

**Fig 1:**
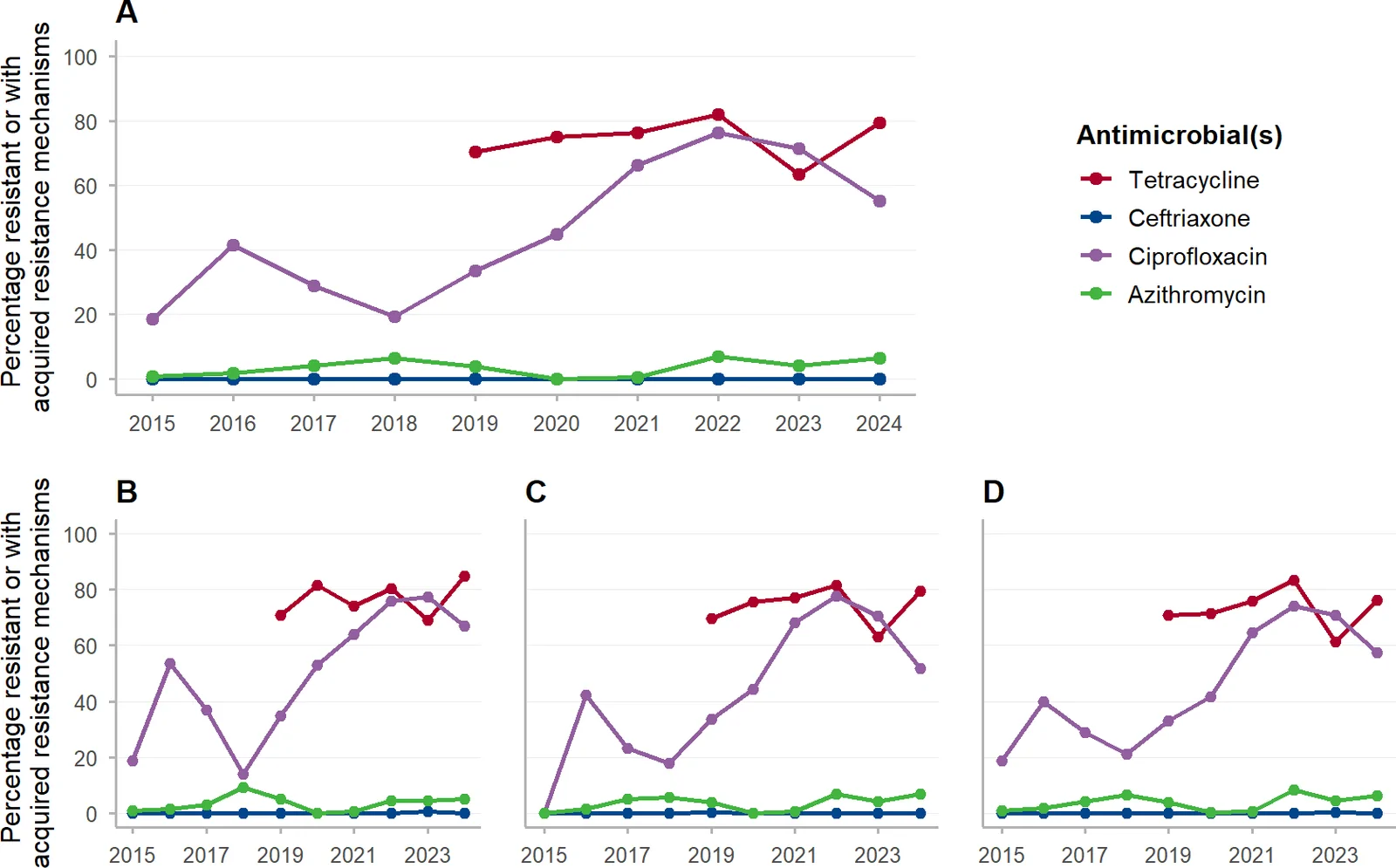
Trends in antimicrobial resistance or acquired resistance mechanisms among *Neisseria gonorrhoeae* isolates from men who have sex with men at Melbourne Sexual Health Centre, 2015–2024. (**A**) All isolates (*n* = 11,334). (**B**) People living with HIV (*n* = 1,491). (**C**) HIV-negative, PrEP users (*n* = 4,377). (**D**) HIV-negative, non-PrEP users (*n* = 5,466). Tetracycline resistance data are presented from 2019 onward. Isolates tested between 2015 and 2018 had minimum inhibitory concentrations (MICs) ≤4 mg/LThis figure has a comment. See if it is exactly the same with the second figure. , which cannot be classified as susceptible or resistant according to the 2025 European Committee on Antimicrobial Susceptibility Testing (EUCAST) guidelines. For azithromycin, which lacks EUCAST clinical breakpoints, the proportion reflects the proportion of isolates classified as non-wild type (MIC >1 mg/L) based on the epidemiological cutoff value (ECOFF). Sample sizes represent all isolates tested, as multiple anatomical sites may be sampled from a single individual. Cochran-Armitage *P*_trend_ values for overall trends (panel **A**): ceftriaxone *P* = 0.313, azithromycin *P* < 0.001, ciprofloxacin *P* < 0.001, tetracycline *P* = 0.052. *P*_trend_ values stratified by HIV/PrEP status are presented in Table S1.

**Fig 2:**
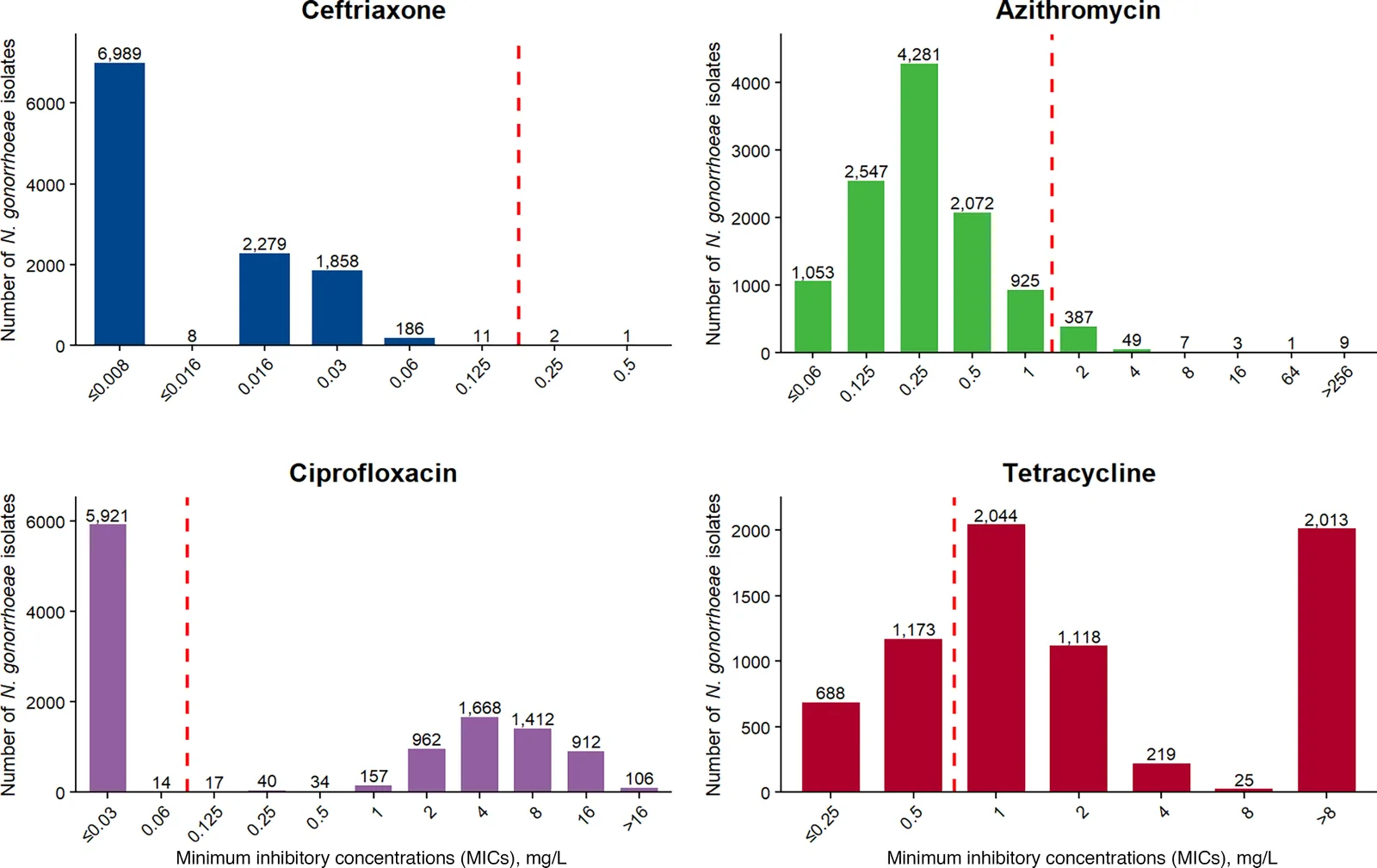
Distribution of minimum inhibitory concentrations (MICs) for ceftriaxone, azithromycin, ciprofloxacin, and tetracycline among N*eisseria gonorrhoeae* isolates from men who have sex with men at Melbourne Sexual Health Centre, 2015–2024. Ceftriaxone and azithromycin distributions are based on isolates collected from 2015 to 2024 (*N* = 11,334). Ciprofloxacin distribution is based on 11,243 isolates, as 91 isolates were not tested for ciprofloxacin. Tetracycline distribution is based on isolates collected from 2019 to 2024 (*n* = 7,280), as MICs recorded between 2015 and 2018 were censored at ≤4 mg/L and could not be classified. Dashed red lines indicate the resistance breakpoints based on the 2025 European Committee on Antimicrobial Susceptibility Testing (EUCAST) guidelines for ceftriaxone, ciprofloxacin, and tetracycline. For azithromycin, the dashed red line indicates the epidemiological cutoff value (ECOFF, MIC >1 mg/L), as EUCAST clinical breakpoints are not available.

The proportion of isolates with acquired azithromycin resistance mechanisms was 4.0% (456/11,334), but it increased significantly from 0.9% (8/940) in 2015 to 6.4% (102/1,584) in 2024 (*P*_trend_ < 0.001) (Fig. 1 and 2). Of these, nine (2.0%) had highly elevated azithromycin MICs (MIC ≥256 mg/L). Ciprofloxacin resistance showed the most pronounced change, increasing from 18.5% (170/920) in 2015 to 55.2% (875/1,584) in 2024 (*P*_trend_ < 0.001). Tetracycline resistance, assessable from 2019 onward, was consistently high, ranging from 70.5% (874/1,240) in 2019 to 79.4% (1,257/1,584) in 2024, with no significant trend (*P*_trend_ = 0.052). The MIC distribution was bimodal, with peaks at 1 mg/L and above 8 mg/L (Fig. 1 and 2).

### Trends stratified by HIV and PrEP status

Resistance patterns were broadly similar across the three groups (Fig. 1B through D; Tables S1 and S2A). A significant rising trend in azithromycin non-wild-type isolates was observed among PrEP users (*P*_trend_ = 0.001) and non-PrEP users (*P*_trend_ < 0.001), but not among PLHIV (*P*_trend_ = 0.293), although the overall proportion did not differ significantly between groups (*P* = 0.063). Ciprofloxacin resistance increased significantly in all three groups (all *P*_trend_ < 0.001), reaching 67.0% (132/197) among PLHIV, 57.5% (245/426) among non-PrEP users, and 51.8% (498/961) among PrEP users by 2024. Overall, ciprofloxacin resistance was significantly higher among PrEP users (54.6%, 2,367/4,334) and PLHIV (50.8%, 755/1,485) than non-PrEP users (40.3%, 2,186/5,424; *P* < 0.001). Tetracycline resistance was high across all groups from 2019 onward (range 72.8% [1,873/2,571] among non-PrEP users to 76.9% [765/995] among PLHIV; *P* = 0.032), with no significant trends in any subgroup (all *P*_trend_ > 0.1).

### AMR by country of birth and anatomical site

**Fig 3:**
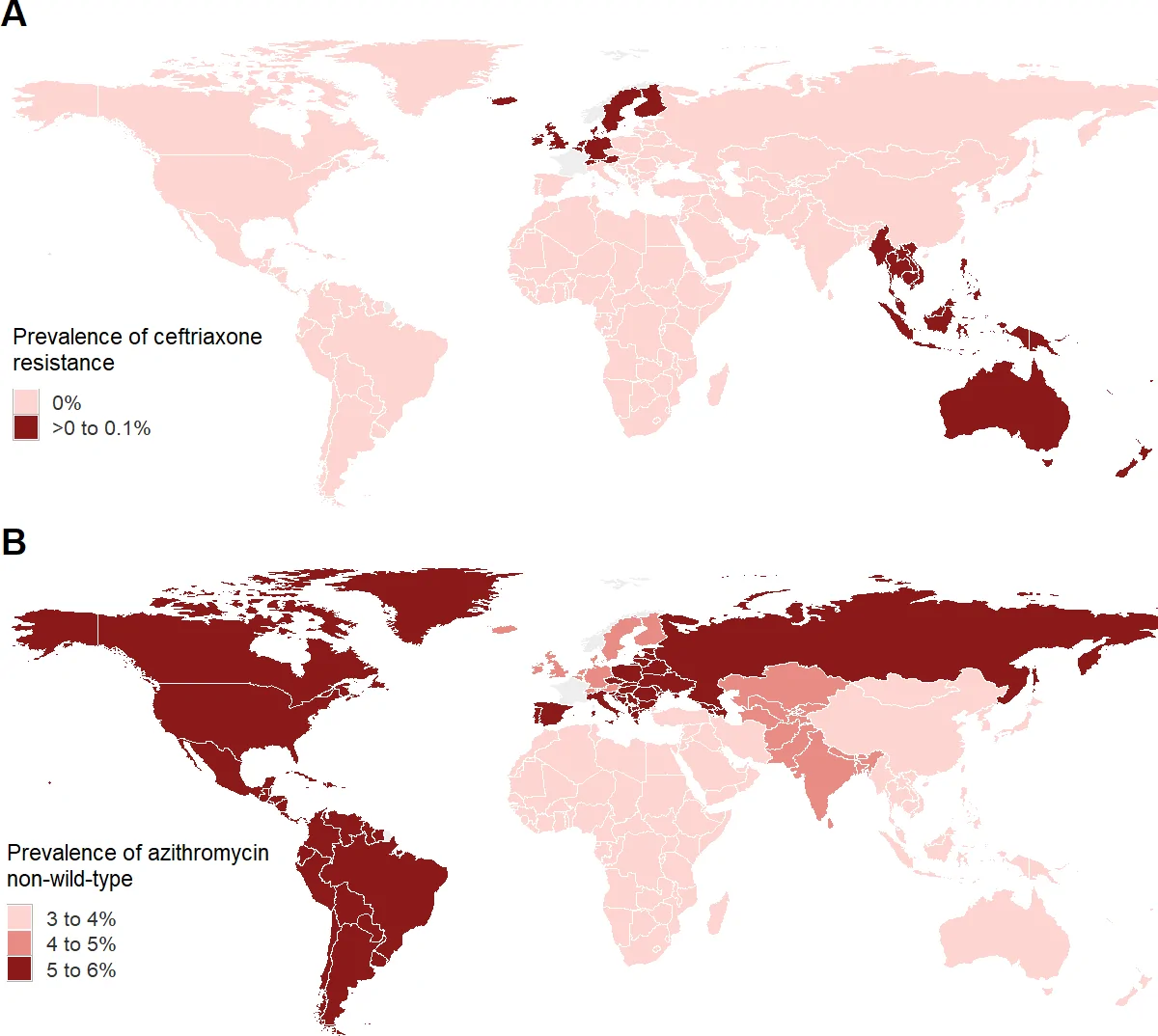
Proportion of ceftriaxone resistance and azithromycin non-wild-type isolates of *Neisseria gonorrhoeae* among men who have sex with men, by country of birth, Melbourne Sexual Health Centre, 2015–2024. The estimates are based on the country of birth of individuals attending Melbourne Sexual Health Centre and do not represent national resistance rates in those countries. Countries are shaded according to the proportion of ceftriaxone resistance (panel **A**) or azithromycin non-wild-type isolates (panel **B**) among isolates from individuals born in that country. Map boundaries were generated in R using the rnaturalearth package.

**Fig 4:**
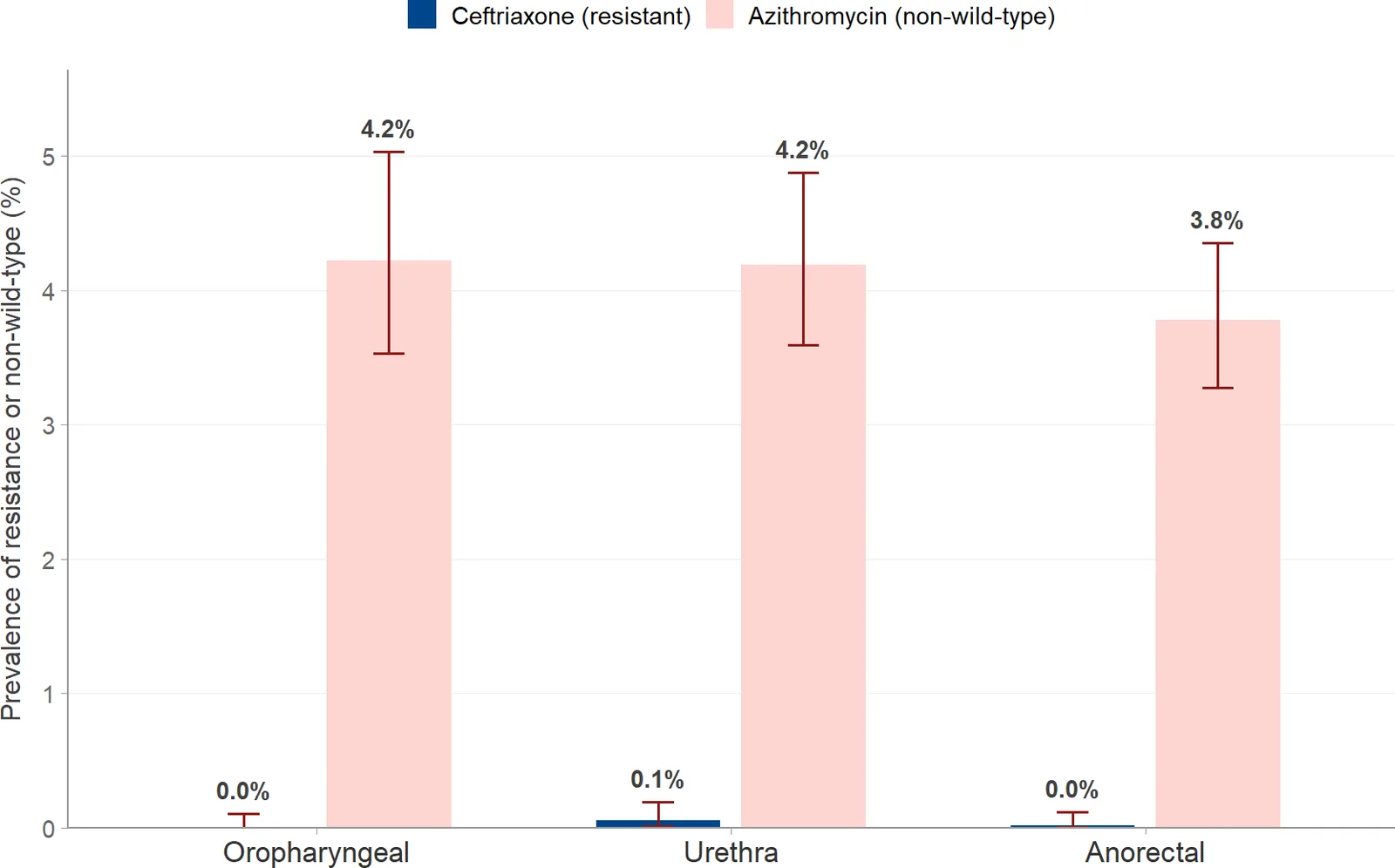
Proportion of ceftriaxone resistance and azithromycin non-wild-type isolates of *Neisseria gonorrhoeae* among men who have sex with men, by anatomical sampling site, Melbourne Sexual Health Centre, 2015–2024 (*N* = 11,334). Error bars represent 95% confidence intervals.

Three ceftriaxone-resistant isolates were detected: one each from individuals born in Australia, Vietnam, and Germany (Fig. 3). The proportion of azithromycin non-wild-type was highest (5–6%) among those born in the Americas, Sub-Saharan Africa, and Northeast Asia. By anatomical site, azithromycin non-wild-type proportion and tetracycline resistance did not differ significantly (all *P* > 0.05; Fig. 4; Table S2B). Ciprofloxacin resistance was highest in oropharyngeal isolates (49.0%, 1,346/2,746), followed by anorectal (48.0%, 2,293/4,776) and urethral (44.9%, 1,668/3,719) isolates (*P* = 0.027).

### Tetracycline resistance and doxyPEP use

Of the 1,668 isolates collected from December 2023 onward, 1,020 had corresponding data on doxyPEP use, including 120 (11.8%) from doxyPEP users and 900 (88.2%) from non-doxyPEP users. Tetracycline resistance was significantly higher among doxyPEP users than non-doxyPEP users (89.2% [107/120] vs 77.2% [695/900], *P* = 0.003; Table 2).

**TABLE 2:** Tetracycline resistance among *Neisseria gonorrhoeae* isolates by doxyPEP use, Melbourne Sexual Health Centre, 2023–2024*^a^^,^^b^*

DoxyPEP use	Resistant	Susceptible	Total	% Resistant
Yes	107	13	120	89.2%
No	695	205	900	77.2%
Total	802	218	1,020	78.6%

^*a*^ Pearson’s *χ*² = 8.99, *P* = 0.003.

^*b*^ DoxyPEP use was recorded through the Digital Front Door (DFD) system, available from December 2023 onward.

When tetracycline resistance was further stratified by MIC level, 54.2% (65/120) of isolates from doxyPEP users had high-level resistance (MIC >8 mg/L) compared with 29.7% (267/900) among non-users (*P* < 0.001). High-level resistant isolates were significantly more likely to be co-resistant to ciprofloxacin (86.0%, 1,732/2,013) than low-level resistant (60.3%, 2,054/3,406) or susceptible (26.3%, 489/1,861) isolates (*P* < 0.001; Table S3).

## DISCUSSION

In this 10-year analysis of 11,334 *N. gonorrhoeae* isolates from MSM in Melbourne, we identified several important trends in AMR. Ceftriaxone resistance remained rare. However, three resistant isolates were detected, and two of these had resistance profiles consistent with those of extensively drug-resistant strains that have been reported internationally. The proportion of isolates with acquired azithromycin resistance mechanisms increased from 0.9% to 6.4%, now exceeding the 5% population resistance threshold deemed acceptable for an antimicrobial to be recommended for empirical management by the WHO (25). Ciprofloxacin resistance increased from 18.5% to 55.2%, reversing the downward trend previously reported at this clinic between 2007 and 2018 (20). Tetracycline resistance was consistently high and was significantly higher among doxyPEP users than non-users. These findings have two main implications for gonorrhea management. First, the rising proportion of azithromycin non-wild-type isolates raises questions about the continued use of dual ceftriaxone-azithromycin therapy in Australia. Second, tetracycline resistance was higher among doxyPEP users. Tetracycline is not currently recommended to treat gonorrhea, so this does not directly affect treatment. It does, however, highlight the need to monitor for multidrug-resistant *N. gonorrho*eae as doxyPEP use expands.

The increasing proportion of azithromycin non-wild-type isolates (i.e., isolates with acquired azithromycin resistance mechanisms, MIC >1 mg/L, above the ECOFF) is a clinically significant finding of this study. Azithromycin remains a component of the recommended dual therapy for gonorrhea in Australia (6). A previous study from the same sexual health clinic reported azithromycin resistance of 4.9% among isolates tested in 2021–2022, raising early concerns about the efficacy of dual therapy (3).

The proportion of non-wild-type isolates exceeded 5%, with 7.0% in 2022 and 6.4% in 2024. The WHO recommends that an antimicrobial should no longer be used for empirical treatment once resistance exceeds 5% (25). This threshold refers to clinical resistance, whereas our estimate is based on the ECOFF. The two are therefore not directly equivalent, and our findings should be read as a signal of rising acquired resistance rather than as a direct measure of clinical resistance. This rise in non-wild-type isolates was driven primarily by PrEP users and non-PrEP users, although the overall proportion did not differ significantly between HIV and PrEP groups. Azithromycin exposure through gonorrhea dual therapy itself, as well as its historical use as first-line treatment for chlamydia, likely exerts bystander selective pressure on co-colonizing *N. gonorrhoeae* (26), contributing to the rising proportion of non-wild-type isolates among MSM undergoing high-frequency STI screening. Several countries, including the United States and the United Kingdom, have already moved away from dual therapy in favor of ceftriaxone monotherapy, partly in response to rising azithromycin resistance and antimicrobial stewardship concerns (7, 8, 27, 28). Our findings reinforce the concerns raised previously (3) about the long-term reliability of azithromycin as a combination partner in dual therapy and support the need for ongoing review of gonorrhea treatment recommendations in Australia.

The substantial rise in ciprofloxacin resistance from 18.5% in 2015 to 55.2% in 2024 is notable as it represents a reversal of the earlier trend observed at MSHC. Our previous study, covering 2007 to 2018, reported a decline in ciprofloxacin resistance from 63.5% to 21.1% among MSM at the same clinic (20). This reversal was consistent across all three HIV and PrEP groups, suggesting that the drivers are not specific to any particular subpopulation. Possible explanations include changes in sexual mixing patterns, increased international travel after the COVID-19 pandemic, and the spread of ciprofloxacin-resistant lineages within domestic sexual networks (29). Nationally, the 2024 Australian Gonococcal Surveillance Programme reported ciprofloxacin resistance of 57.5% (10), close to the 55.2% observed in our study. A similar upward trend has been observed across Europe, where ciprofloxacin resistance reached 65.9% in 2022 (30). In the WHO Western Pacific Region, ciprofloxacin resistance was substantially higher at 95.3% across nine sentinel countries in 2023 (31). Historically, the establishment of ciprofloxacin resistance in Australia has been attributed to the repeated importation of multiple resistant genotypes, predominantly from Asia (32). In 2022, the rapid expansion of the ciprofloxacin-resistant ST-7827 clone in New South Wales contributed to a further increase nationally (10, 33). In our study, 12.3% of individuals were born in Southeast Asia, and 12.0% reported overseas sexual contact in the past 12 months. Country of birth is a proxy variable associated with significant variation in gonococcal AMR risk (20), which may reflect several mechanisms: individuals born in countries with a higher background prevalence of gonococcal AMR may have been exposed to resistant strains before migration, and differences in antibiotic access, antimicrobial stewardship practices, and the circulation of resistant strains across countries may also contribute to the observed variation. Additionally, ongoing travel and sexual networks may facilitate continued importation of resistant strains. However, genomic characterization of isolates would be needed to confirm the relative contributions of importation and domestic transmission. Ciprofloxacin has not been recommended for empirical gonorrhea treatment for many years due to high resistance levels, but it has been used as resistance-guided therapy (RGT) following culture and susceptibility testing. With ciprofloxacin resistance now exceeding 55% among MSM in our setting, the proportion of patients eligible for ciprofloxacin-based RGT is substantially reduced, limiting its feasibility as a treatment strategy. Recent guidelines from the WHO (9) and the United Kingdom (7) have also moved away from ciprofloxacin-based RGT, citing both high rates of resistance and safety concerns associated with fluoroquinolone use.

The finding of significantly higher tetracycline resistance among doxyPEP users compared with non-users is consistent with emerging evidence from clinical trials and observational studies. The ANRS 174 DOXYVAC trial reported that high-level tetracycline resistance was nearly three times more common in the doxyPEP arm compared with the no-doxyPEP arm (34), and the U.S. doxyPEP trial similarly demonstrated increased tetracycline resistance at follow-up among doxyPEP users (35). Our study extends these findings to a real-world, non-trial setting with over 1,000 isolates, making it one of the largest observational comparisons of tetracycline resistance by doxyPEP status reported to date. Notably, background tetracycline resistance was already high (77.2%) even among non-doxyPEP users, which may partly explain the more modest effect of doxyPEP on gonorrhea prevention compared with its efficacy against syphilis and chlamydia observed in clinical trials (36). As doxyPEP use expands in Australia following the 2023 ASHM consensus statement, early data from a national survey conducted in 2024 showed that around 15% of MSM in Australia had ever used doxyPEP (37). Although tetracycline is not currently recommended for the treatment of gonorrhea because of widespread resistance, and is not routinely tested in many surveillance programs (38), it provides a direct marker of the selective pressure exerted by doxyPEP on *N. gonorrhoeae*. The broader impact of doxyPEP on AMR may extend beyond *N. gonorrhoeae* to other pathogens, including *Staphylococcus aureus*, *Treponema pallidum*, *Chlamydia trachomatis*, and commensal flora. For *T. pallidum* and *C. trachomatis*, this remains a theoretical concern, as doxycycline resistance has not been demonstrated in either organism to date. Ongoing monitoring will be needed as doxyPEP use expands. Moreover, doxyPEP users had a higher proportion of high-level tetracycline resistance (MIC >8 mg/L; 54.2% vs 29.7%), which likely reflects plasmid-mediated resistance. Across all isolates from 2019 to 2024, high-level tetracycline resistance was associated with ciprofloxacin co-resistance (86.0%). This is an association between resistance phenotypes, and our study was not designed to assess whether doxyPEP use itself is associated with resistance to other antimicrobials.

Although ceftriaxone decreased susceptibility/resistance remained rare in our study, the detection of three resistant isolates over the past decade requires attention. Nationally, the AGSP reported that 0.51% of isolates in 2022 and 0.22% in 2023 had ceftriaxone MIC ≥0.125 mg/L, with an increasing number of XDR isolates being detected across Australia (10). Internationally, ceftriaxone resistance has risen sharply in parts of the WHO Western Pacific Region, with the 2023 WHO EGASP reporting resistance of 15.3% (42/274) in Cambodia and 20.4% (53/260) in Vietnam, where all 95 ceftriaxone-resistant isolates across nine sentinel countries were detected (31). Given the proportion of individuals in our study born in Southeast Asia and those reporting recent overseas sexual contact, importation of ceftriaxone-resistant strains through sexual networks remains a plausible risk that underscores the importance of sustained culture-based surveillance and, where feasible, genomic characterization of resistant isolates.

This study has several strengths. To the best of our knowledge, it is the first to stratify AMR trends among MSM by HIV status and PrEP use over a full decade, including the doxyPEP era. The integration of individual-level demographic, behavioral, and clinical data with laboratory MIC data from over 11,000 isolates provides a depth of detail not available from most national surveillance programs, which typically report aggregated resistance data without patient-level metadata. The use of standardized antimicrobial susceptibility testing performed at a single reference laboratory (MDU PHL) throughout the study period ensures consistency in MIC determination. Finally, the doxyPEP analysis, although limited to a 2-year window, represents one of the largest real-world comparisons of tetracycline resistance by doxyPEP status.

Several limitations should be acknowledged. First, this is a single-center study from a metropolitan public sexual health clinic in Melbourne, and findings may not be generalizable to other settings or populations. However, even in a state-wide gonococcal surveillance program, the majority of gonococcal isolates are derived from sexual health clinics, as routine culture-based testing in general practice is uncommon due to practical constraints (29). In addition, only culture-positive infections were included in this study. As cultures are taken from people recalled after a positive NAAT and from those with symptoms, our isolates likely over-represent symptomatic infections and those with a higher bacterial load. They may therefore not reflect all gonorrhea diagnosed at the clinic. Second, the doxyPEP analysis was limited to the period from December 2023 onward, and the number of doxyPEP users was relatively small (*n* = 120). DoxyPEP use was self-reported through the CASI questionnaire and may therefore be subject to recall or social desirability bias, potentially leading to misclassification of exposure status. Responses were also carried forward for up to 90 days, so some participants may have changed their doxyPEP use in between. This would most likely reduce rather than exaggerate the observed difference. Third, the cross-sectional design of the doxyPEP analysis does not allow us to determine causality. The higher tetracycline resistance among doxyPEP users may reflect the selection of pre-existing resistant strains that were not prevented by doxycycline (breakthrough infections), rather than the induction of *de novo* resistance through antibiotic exposure. Distinguishing between these mechanisms would require longitudinal follow-up and genomic characterization of isolates. Fourth, tetracycline MIC data from before 2019 were recorded as ≤4 mg/L, before a downward shift in breakpoints, which prevented assessment of resistance trends before that year. Fifth, demographic characteristics were summarized once per individual using their first visit, whereas all AMR analyses used HIV and PrEP status at the time each isolate was collected. Individuals whose status changed during the study period therefore appear in a single group in Table 1 but may contribute isolates to more than one group in the AMR analyses. The two sets of group totals are not directly comparable. Finally, this study did not include genomic analysis of resistant isolates, which would provide additional insight into the molecular mechanisms and transmission dynamics of resistance.

In conclusion, ceftriaxone remains effective for empirical gonorrhea treatment among MSM in Melbourne, although the emergence of extensively drug-resistant isolates highlights the ongoing threat of importation. The rising proportion of azithromycin non-wild-type isolates, now above 5%, raises concerns about the utility of azithromycin in dual therapy and as an alternative agent. Ciprofloxacin resistance has reversed its earlier downward trend, further limiting treatment options. Higher tetracycline resistance among doxyPEP users, particularly high-level tetracycline resistance, underscores the need to monitor for multidrug-resistant strains as doxyPEP use expands. These findings should inform gonorrhea treatment guidelines and doxyPEP implementation strategies in Australia and internationally.

## Data Availability

All data generated or analyzed during this study are included in this published article. The data that support this study cannot be publicly shared due to ethical or privacy reasons. Data will be shared upon reasonable request to the corresponding author with permission from the Alfred Hospital Ethics Committee.

## SUPPLEMENTAL MATERIAL

Supplemental Tables jcm.00965-26-s0001.docx Tables S1 to S3.

ASM does not own the copyrights to Supplemental Material that may be linked to, or accessed through, an article. The authors have granted ASM a non-exclusive, world-wide license to publish the Supplemental Material files. Please contact the corresponding author directly for reuse.
