# Supplementary material for: Antimicrobial resistance in Neisseria gonorrhoeae among men who have sex with men: a 10-year analysis with implications for doxycycline post-exposure prophylaxis
Source: J Clin Microbiol. 2026 Sep 25;64(10):e00965-26. doi: 10.1128/jcm.00965-26 (PMC13712797; doi:10.1128/jcm.00965-26)
Supplement: Supplementary file 1 [file jcm.00965-26-s0001.docx]

**Supplementary Tables**

*Table S1. Cochran-Armitage P_trend_ values for antimicrobial resistance trends among Neisseria gonorrhoeae isolates from men who have sex with men, overall and stratified by HIV and PrEP status, Melbourne Sexual Health Centre, 2015-2024*

|  | **Ceftriaxone** | **Azithromycin^†^** | **Ciprofloxacin** | **Tetracycline^‡^** |
| --- | --- | --- | --- | --- |
| **Overall (n=11,334)** | 0.313 | <0.001 | <0.001 | 0.052 |
| **People living with HIV (n=1,489)** | 0.287 | 0.293 | <0.001 | 0.101 |
| **HIV-negative, PrEP users (n=4,377)** | 0.288 | 0.001 | <0.001 | 0.169 |
| **HIV-negative, non-PrEP users (n=5,468)** | 0.147 | <0.001 | <0.001 | 0.762 |

† For azithromycin, which lacks EUCAST clinical breakpoints, trends refer to the proportion of non-wild-type isolates (MIC >1 mg/L) based on the epidemiological cut-off value (ECOFF).

‡ Tetracycline resistance was assessed from 2019 onwards only. Isolates tested between 2015 and 2018 had MICs ≤4 mg/L, which did not permit classification as susceptible or resistant.

Sample sizes represent all isolates tested, as multiple anatomical sites may be sampled from a single individual. Bold values indicate P<0.05.

*Table S2. Prevalence of azithromycin non-wild-type isolates and tetracycline resistance among Neisseria gonorrhoeae isolates from men who have sex with men, by HIV and PrEP status and anatomical site, Melbourne Sexual Health Centre, 2015-2024*

**(A) By HIV and PrEP status**

|  | HIV positive  n (%) | PrEP  users  n (%) | Non-PrEP  users  n (%) | Total  n (%) | P-value |
| --- | --- | --- | --- | --- | --- |
| Azithromycin non-wild-type^†^ | 54/1491 (3.6%) | 200/4377 (4.6%) | 202/5466 (3.7%) | 456/11334 (4.0%) | 0.063 |
| Ciprofloxacin resistant | 755/1485 (50.8%) | 2367/4334 (54.6%) | 2186/5424 (40.3%) | 5308/11243 (47.2%) | <0.001 |
| Tetracycline resistant^‡^ | 765/995 (76.9%) | 2781/3714 (74.9%) | 1873/2571 (72.8%) | 5419/7280 (74.4%) | 0.032 |

**(B) By anatomical site**

|  | Anorectum  n (%) | Oropharynx n (%) | Urethra  n (%) | Total  n (%) | P-value |
| --- | --- | --- | --- | --- | --- |
| Ceftriaxone resistant | 1/4814 (0.02%) | 0/2771  (0%) | 2/3747 (0.05%) | 3/11332 (0.03%) | 0.615* |
| Azithromycin non-wild-type^†^ | 182/4814 (3.8%) | 117/2771 (4.2%) | 157/3747 (4.2%) | 456/11332 (4.0%) | 0.712 |
| Ciprofloxacin resistant | 2293/4776 (48.0%) | 1346/2746 (49.0%) | 1668/3719 (44.9%) | 5307/11,241 (47.2%) | 0.027 |
| Tetracycline resistant^‡^ | 2386/3173 (75.2%) | 1295/1728 (74.9%) | 1738/2378 (73.1%) | 5419/7279 (74.4%) | 0.094 |

† For azithromycin, which lacks EUCAST clinical breakpoints, the proportion reflects non-wild-type isolates (MIC >1 mg/L) based on the epidemiological cut-off value (ECOFF).

‡ Tetracycline resistance assessed from 2019 onwards only.

* Fisher's exact test used due to small expected cell counts; Pearson chi-squared test used for all other comparisons.

Isolates classified as 'Others' (n=2) were excluded from the anatomical site analysis. Ciprofloxacin isolates classified as 'susceptible, increased exposure' (n=14) were not included in the resistant category.

*Table S3. Tetracycline resistance subcategories among Neisseria gonorrhoeae isolates from men who have sex with men, by year, HIV and PrEP status, doxyPEP use, and ciprofloxacin co-resistance, Melbourne Sexual Health Centre, 2019-2024*

**(A) By year**

| **Year** | **Susceptible**  **n (%)** | **Low-level resistant**  **(MIC 1-8 mg/L) n (%)** | **High-level resistant**  **(MIC >8 mg/L)**  **n (%)** | **Total** |
| --- | --- | --- | --- | --- |
| 2019 | 366 (29.5%) | 641 (51.7%) | 233 (18.8%) | 1,240 |
| 2020 | 204 (25.1%) | 440 (54.1%) | 170 (20.9%) | 814 |
| 2021 | 221 (23.8%) | 513 (55.2%) | 195 (21.0%) | 929 |
| 2022 | 244 (18.1%) | 685 (50.7%) | 421 (31.2%) | 1,350 |
| 2023 | 499 (36.6%) | 435 (31.9%) | 429 (31.5%) | 1,363 |
| 2024 | 327 (20.6%) | 692 (43.7%) | 565 (35.7%) | 1,584 |

**(B) By HIV and PrEP status**

| **HIV/PrEP**  **Status** | **Susceptible**  **n (%)** | **Low-level resistant**  **(MIC 1-8 mg/L) n (%)** | **High-level resistant**  **(MIC >8 mg/L) n (%)** | **Total** |
| --- | --- | --- | --- | --- |
| HIV positive | 230 (23.1%) | 438 (44.0%) | 327 (32.9%) | 995 |
| PrEP users | 933 (25.1%) | 1,744 (47.0%) | 1,037 (27.9%) | 3,714 |
| Non-PrEP users | 698 (27.2%) | 1,224 (47.6%) | 649 (25.2%) | 2,571 |

**(C) By DoxyPEP use**

| **DoxyPEP**  **Use** | **Susceptible n (%)** | **Low-level resistant (MIC 1-8 mg/L) n (%)** | **High-level resistant (MIC >8 mg/L) n (%)** | **Total** |
| --- | --- | --- | --- | --- |
| Users | 13 (10.8%) | 42 (35.0%) | 65 (54.2%) | 120 |
| Non-users | 205 (22.8%) | 428 (47.6%) | 267 (29.7%) | 900 |
| Total | 218 (21.4%) | 470 (46.1%) | 332 (32.5%) | 1,020 |

Pearson chi-squared test: p<0.001

**(D) Ciprofloxacin co-resistance by tetracycline resistance category**

| **Tetracycline category** | **Ciprofloxacin resistant**  **n (%)** | **Azithromycin non-wild-type^†^ n (%)** | **Total** |
| --- | --- | --- | --- |
| Susceptible | 489 (26.3%) | 33 (1.8%) | 1,861 |
| Low-level resistant (MIC 1-8) | 2,054 (60.3%) | 261 (7.7%) | 3,406 |
| High-level resistant (MIC >8) | 1,732 (86.0%) | 7 (0.3%) | 2,013 |

Pearson chi-squared test: p<0.001

Low-level tetracycline resistance (MIC 1-8 mg/L) is more likely to reflect chromosomally mediated mechanisms, whereas high-level resistance (MIC >8 mg/L) is more likely to reflect plasmid-mediated mechanisms (tetM gene).

DoxyPEP use was recorded through the Digital Front Door (DFD), available from December 2023 onwards.

^†^ For azithromycin, which lacks EUCAST clinical breakpoints, the proportion reflects non-wild-type isolates (MIC >1 mg/L) based on the epidemiological cut-off value (ECOFF).
